# Research Progress of Universal Influenza Vaccine

**DOI:** 10.3390/vaccines13080863

**Published:** 2025-08-15

**Authors:** Liangliang Wang, Qian Xie, Pengju Yu, Jie Zhang, Chenchen He, Weijin Huang, Youchun Wang, Chenyan Zhao

**Affiliations:** 1 Chinese Academy of Medical Sciences & Peking Union Medical College, Beijing 100730, China; wanglliang3@mail2.sysu.edu.cn; 2 Division of HIV/AIDS and Sex-transmitted Virus Vaccines, Institute for Biological Product Control, National Institutes for Food and Drug Control (NIFDC), Beijing 102629, China; yupengju@nifdc.org.cn (P.Y.); zhangjie2024@nifdc.org.cn (J.Z.); chench07@outlook.com (C.H.); huangweijin@nifdc.org.cn (W.H.); 3 WHO Collaborating Center for Standardization and Evaluation of Biologicals, Beijing 102629, China; 4 NHC Key Laboratory of Research on Quality and Standardization of Biotech Products and NMPA Key Laboratory for Quality Research and Evaluation of Biological Products, Beijing 102629, China; 5 School of Public Health (Shenzhen), Sun Yat-sen University, Shenzhen 518107, China; xieq57@mail2.sysu.edu.cn

**Keywords:** influenza vaccine, universal vaccine, antigen targets, antigen design strategies

## Abstract

Influenza viruses continue to undergo antigenic drift and shift, resulting in the need to update existing vaccines annually. Therefore, the development of a universal influenza vaccine has become an urgent global need. This paper reviews the functions of common antigenic targets of influenza vaccines and their advantages and disadvantages in universal vaccine design. We also summarize the common design strategies for universal influenza vaccines, which mainly include the immunofocusing strategy, multi-target combination strategy, T-cell strategy, computationally optimized broadly cross-reactive antigenic strategy (COBRA), and artificial intelligence strategy. In addition, we also sort out the latest research progress of universal influenza vaccines under different technological routes. This will help researchers better grasp the latest developments of universal influenza vaccines.

## 1. Introduction

Seasonal epidemics of influenza are estimated to cause 3–5 million severe cases and 290,000–650,000 deaths annually worldwide [1]. Influenza can also lead to pandemics. There have been four pandemics caused by influenza viruses in history [2]. The worst of these was the 1918 influenza pandemic, which caused the deaths of approximately 100 million people [3]. Thus, influenza infections pose a significant burden on global public health. Vaccination is the most effective measure to reduce the disease burden and control infectious diseases. The World Health Organization (WHO) has established the Global Influenza Surveillance and Response System (GISRS) since 1952, which annually recommends vaccine strains for the next seasonal influenza viruses in the southern hemisphere (September) and the northern hemisphere (February) on the basis of antigenic data from ferret sera, serological analyses of human samples, and viral sequence data [4].

Influenza vaccines may vary in effectiveness due to inconsistencies between the prevalent strain and the vaccine strain [5], caused by the rapid mutation of influenza viruses. In addition, the use of chicken embryos to produce seasonal influenza vaccines can lead to slow production rates and reduce the effectiveness of the vaccine when adaptive mutations occur in chicken embryos [6,7]. Furthermore, emerging influenza pandemics render seasonal influenza vaccines virtually ineffective, and a general lack of immunity in the population exacerbates symptoms. As a result, there is an urgent need to develop an effective universal influenza vaccine. Based on a description of influenza virus variation and the standard definition of a universal influenza vaccine, this paper summarizes the antigenic targets and antigenic design strategies commonly used for universal influenza vaccines and provides a detailed summary of the latest advances in universal influenza vaccines of different technological routes in recent years.

## 2. Influenza Viruses and Criteria for a Universal Influenza Vaccine

### 2.1. Influenza Viruses and Mutations

Influenza viruses belong to the Orthomyxoviridae family and are single-stranded, negative-stranded RNA viruses [8]. The viral genome is divided into a total of eight segments encoding a total of more than a dozen proteins, mainly including haemagglutinin (HA), neuraminidase (NA), nuclear protein (NP), two matrix proteins (M1 and M2), acidic polymerase (PA), two alkaline polymerase proteins (PB1 and PB2), and non-structural protein (NS1) [9]. Based on the antigenic differences of NP and M1, influenza viruses can be classified into four types: A, B, C, and D. Influenza A and B viruses cause seasonal epidemics mainly in humans. Influenza A viruses are the main cause of influenza pandemics [2]. Influenza A viruses are classified into 18 HA subtypes (H1–H18) and 11 NA subtypes (N1–N11) based on the sequence similarity between HA and NA [10]. HA subtypes are further classified into Group I and Group II based on the similarity of HA. There are only two lineages of influenza B viruses: the Victoria lineage and the Yamagata lineage.

Influenza viruses mutate mainly through antigenic drift and antigenic shift. Antigenic drift refers to the accumulation of point mutations in viral proteins due to the lack of proofreading activity of the RNA polymerase of influenza viruses, or the glycosylation of viral proteins [11,12], which ultimately leads to changes in antigenicity. Seasonal antigenic drift contributes to seasonal epidemics of influenza, as well as explaining the need for annual renewal of seasonal influenza vaccines. Antigenic shift refers to the rearrangement of fragments between viral genomes that occurs when different influenza viruses infect the same host [13]. Through antigenic shift new subtypes or new strains may be created and the population often lacks immunity to these new subtypes and strains, leading to pandemic influenza.

### 2.2. Standards for Universal Influenza Vaccine

For seasonal pandemics of influenza, while seasonal influenza vaccines are available on the market, they often need to be updated annually. For potential pandemic influenza, there is often no timely available vaccine. Therefore, there is a need to develop a universal vaccine. Different organizations have defined different standards for a universal influenza vaccine. The National Institute of Allergy and Infectious Diseases (NIAID), a part of the National Institutes of Health (NIH), in June 2017 set the criteria for a universal influenza vaccine [14,15]: (1) protection against at least 75% of symptomatic influenza infections; (2) work in all age groups; (3) protection lasts more than 1 year or spans more than 1 season; and (4) effectiveness against both Group I and Group II influenza A are both effective, or also for influenza B. The World Health Organization has also set out new standards for universal flu vaccines in 2024: (1) work in all age groups; (2) protection against a minimum of 3 years for current circulating subtypes; (3) protection better than currently approved seasonal influenza vaccines for currently circulating subtypes.

## 3. Antigenic Targets and Design Strategies for Universal Influenza Vaccine

### 3.1. Antigenic Targets of Universal Influenza Vaccines

HA, NA, NP, M1, and M2 are the most common antigenic targets for universal influenza vaccine design (Table 1). HA is a type I transmembrane glycoprotein on the surface of influenza viruses and exists as a homotrimer, with each monomer consisting of a variable globular head and a more conserved stem. Antibodies directed against the head of the HA neutralize viral infection by preventing binding to the receptor and are usually strain-specific [16]. Antibodies directed against the HA stem can inhibit viral entry and viral release, and are usually cross-protective against various strains and subtypes [17]. However, the level of antibodies against the HA stem is usually inferior to those produced against the HA head, and needs to be increased by stabilizing the conformation and multiple immunizations [18,19,20]. Therefore, there is a need to overcome the disadvantage of immune subdominance of the HA stem when selecting it as a target for a universal influenza vaccine.

NA is a type II transmembrane glycoprotein on the surface of influenza viruses and exists as a homotetramer. The primary function of NA is to cleave the salivary acid receptor to promote the release of progeny viruses [21], so antibodies against NA may provide protection by blocking the release of progeny viruses or through antibody-dependent cellular cytotoxicity (ADCC). NA is antigenically more conserved than HA and anti-NA antibodies can induce protection against both homotypic and subtypic influenza viruses [22]. Therefore, when selecting NA as a target for a universal influenza vaccine, although NA is unable to produce neutralizing antibodies, it can often induce broad-spectrum immune protection.

M2 is a type III transmembrane protein on the surface of influenza viruses and exists as a homotetramer. The primary function of M2 is to facilitate viral entry and exit by controlling pH in endosomes and the Golgi apparatus [23,24,25]. The 23 amino acids at the N-terminal end of M2 are exposed in the extracellular region, which is highly conserved in influenza viruses and is called the M2 extracellular domain (M2e) [26]. Antibodies against M2e do not prevent viral entry, can kill infected cells by ADCC effects or by recruiting innate immune cells, and can induce good cross-protective immunity in mice [27,28]. However, M2e itself is poorly immunogenic. Therefore, multi-target combinations or adjuvants are often required when selecting M2e as a target for a universal influenza vaccine.

NP and M1 are internal proteins of influenza viruses; M1 contributes to the formation of viral particles and NP binds to viral RNA to form a complex that contributes to viral replication [29]. Both contain highly conserved cytotoxic T-cell epitopes (CTLs) [30]. Antibodies against NP and M1 produce cross-protective immunity against different subtypes of influenza virus [31]. Cytotoxic T-cell responses reduce the rate of severe disease and mortality, but do not prevent infection and usually require concomitant induction of an antibody response to be used [32]. Therefore, when selecting NP or M1 as a target for a universal influenza vaccine, a combination of antigens capable of inducing B-cell immunity is often required. Table 1 summarizes the structure–function of the five common targets of the universal influenza vaccine and the immune responses elicited.

### 3.2. Antigen Design Strategies for Universal Influenza Vaccines

Based on the above antigenic targets, the design of a universal influenza vaccine is mainly carried out by strategies such as the immunofocusing strategy, multi-target combination strategy, T-cell strategy, computationally optimized broadly cross-reactive antigenic strategy (COBRA), and artificial intelligence strategy (AI).

#### 3.2.1. Immunofocusing Strategy

Immunofocusing is a strategy to concentrate the immune response at a key epitope or conserved epitope. Focusing the immune response on the HA stem is a hot topic in the development of universal influenza vaccines because the HA stem sequence is more conserved. Using the ‘head-swapping strategy’, in which the head of an exotic avian influenza HA is transplanted into the stem of a human influenza HA, antibodies against the influenza HA stem can be enhanced, thereby improving the cross-protection of the vaccine [33,34]. Using a ‘structural biology strategy’, in which the HA stem is stabilized by removing the HA head and then structurally modifying the HA stem, cross-neutralizing antibodies can be induced in mice and primates and protect mice against multiple strains of influenza [35]. Using an ‘antigen-flipping strategy’, in which an aluminum adjuvant is bound to the HA head and thus redirects the antigen to the HA stem, it is possible to induce the production of protective antibodies to different subtypes of influenza [36]. In addition, vaccines designed via a ‘mutant library strategy’, which uses a library of mutants at the HA antigenic site, can induce more antibody responses against the HA stem, thereby enhancing the broad-spectrum nature of the vaccine [37]. Nevertheless, the immunofocusing strategy frequently depends on the accurate presentation of the natural conformation of the target epitope. This renders vaccine design particularly challenging for complex conformational epitopes. And the more focused the immunogen is means that the immunogenicity of the immunogen is insufficient.

#### 3.2.2. Multi-Target Combination Strategy

The multi-target combination strategy refers to the mixing of different antigens or the preparation of different antigens onto the same vector with the aim of inducing broad-spectrum antibody levels through a combination strategy. The Stanford team designed a broad-spectrum influenza vaccine by covalently coupling different HA antigens; inoculation of mice with coupled heterologous antigens can limit antibody subtype preference compared to HA mixtures alone [38]. In human tonsillar organoids, coupled heterologous HA antigens stimulated higher antibody responses against multiple subtypes and increased activation of CD4^+^ Tfh cells compared to commercial influenza vaccines or HA mixtures [38]. Notably, a method of co-expressing HA and NA was developed by Duke University, and the application of this method to a seasonal influenza vaccine was designed to elicit not only a strong immune response to a matching strain, but also a more cross-reactive antibody profile against a heterologous virus [39]. In addition, vaccines designed by coupling HA, NA, NP, or M2 into the same vector similarly induced a broad spectrum of antibody responses [40,41]. However, the multi-target combination strategy may result in a bias of the immune response of the vaccine towards one component. The increase in the total amount of antigens due to the multi-target combination may lead to an increased risk of adverse reactions to the vaccine, and the vaccine effect of the multi-target combination strategy is often required to be no less than that of a single component, which increases the complexity of the experimental design and the difficulty of regulatory approval.

#### 3.2.3. T-Cell Strategy

It is evident that T-cell epitopes are characterized by heightened conservation, and that T-cell immune responses are instrumental in the protection of individuals against viral infections. Consequently, researchers frequently employ a T-cell strategy in the design of broad-spectrum vaccines, with the objective of activating the T-cell immune response within. The mosaic algorithm was able to induce superior cross-protective responses in mice by presenting a greater number of T-cell epitopes in the immunogen [42,43]. Computer-designed T-cell epitope-based vaccines have been shown to induce virus-specific T-lymphocyte responses and provide partial protection against different influenza virus subtypes [44]. Furthermore, the influenza NP, M1, and PB1 proteins exhibit a high degree of conservation and are recognized by T-cells. Vaccines that have been designed using NP, M1, and PB1 have been shown to induce broad-spectrum reactive T-cells and have been found to be protective against heterotypic influenza [45]. However, vaccines that rely on a T-cell strategy may not offer complete protection against viral infections, often requiring the contribution of B-cell immunity. The diversity and complexity of the population major histocompatibility complex (MHC) leads to differences in T-cell epitope presentation and hence possible differences in population immunological efficacy.

#### 3.2.4. COBRA Strategy

The COBRA strategy refers to a computational tool that extracts the most conserved parts of an immunogen to form a new antigen to induce a broad antibody response. The strategy is designed to cover antigens from a wide range of viruses, improving the vaccine’s resilience to viral variation. The HA COBRA vaccine against H1 influenza can elicit antibodies against a wide range of H1 strains [46]. The HA COBRA vaccine against H3 influenza had a better antibody response than the wild-type HA vaccine against a wide range of past epidemics of H3 influenza, and more notably, the vaccine designed by this method was able to neutralize future emerging H3 strains [47]. In addition, COBRA vaccines designed against NA induced a wider range of cross-reactive antibodies compared to wild-type NA vaccines [48]. However, the COBRA strategy is overly reliant on viral sequences that already exist in nature, and in the event of novel or reassigned viral strains, its induced antibodies may not be able to neutralize these new strains. Notably, the design of COBRA strategies is frequently tailored to a specific subtype, thereby hindering the attainment of comprehensive protection across subtypes.

#### 3.2.5. Artificial Intelligence Strategy

With the rapid development of artificial intelligence, the role of artificial intelligence in the field of vaccines has become increasingly prominent. Artificial intelligence strategy refers to the use of algorithmic tools such as machine learning or deep learning to rationally design immunogens, predict antigenic epitopes, and prioritize recommended antigens [49,50]. Prediction of influenza antigenicity by deep convolutional neural network (CNN) models for influenza vaccine strain recommendation outperforms the World Health Organization’s recommended models and shows consistently good coverage [51]. The ability to accurately distinguish between antigenic variants and non-variants by machine learning models with the help of HA sequences of H3 influenza isolates and related data is of great significance for the development of a universal influenza vaccine [52]. However, vaccines designed by current AI strategies have increased the difficulty of regulatory approval due to poorly interpretable models, while vaccines designed in this emerging discipline have unknown safety due to a lack of large-volume, long-term clinical data observations.

In summary, these five main strategies reflect their respective strengths and weaknesses in universal vaccine design. The immune-focused, T-cell, and COBRA strategies all focus on more conserved epitopes, whereas the multi-targeted combination strategy focuses on immune dispersion, and the AI strategy focuses on selecting advantageous immunogens or predicting likely future immunogens. In the design of universal influenza vaccines in the future, it is essential to integrate the advantages of multiple strategies and to avoid their disadvantages, with a view to inducing high levels of cross-immune responses.

## 4. Universal Influenza Vaccines Under Different Technological Routes

Based on the above antigenic targets and antigen design strategies, researchers have carried out the development of universal influenza vaccines under different technology routes. The main technology routes include the following: influenza virus-based technology platform, viral vector-based technology platform, virus-like particle-based technology platform (VLP), nanoparticle-based technology platform, recombinant protein-based technology platform, and nucleic acid-based technology platform.

### 4.1. Universal Influenza Vaccine Based on Influenza Virus

The two main types of influenza virus-based vaccines are inactivated and live attenuated vaccines. Conventional inactivated influenza vaccines based on the full-length of HA are difficult to induce a broad immune response, but the inactivated vaccine obtained by the Duke University team focusing on the HA stem provoked a cross-protective response in mice [53] (Table 2). In the development of traditional attenuated influenza vaccines, influenza strains of non-human origin are rarely used as vaccine strains for vaccinating humans. Interestingly, an attenuated H3N8 influenza vaccine of equine origin was used by a team from Iowa State University to induce neutralizing antibodies against seasonal and highly pathogenic influenza in mice and showed immunoprotection in a variety of hosts [54], which is promising for use in the control of influenza viruses that spread across species (Table 2). This suggests that influenza virus strains of other species origins can also be used as vaccine strains for the universal influenza vaccine. In addition, the Chinese Academy of Sciences team developed a live attenuated influenza vaccine based on proteolysis-targeting chimeric (PROTAC) technology, which induced potent and broad-based humoral, mucosal, and cellular immunity against homologous and heterologous influenza viruses in mice and ferret models [55] (Table 2). This is a major innovative technology in live attenuated influenza vaccines after cold-adapted influenza strains [56], interferon-sensitive strains [57], and ΔNS1 strains [58]. Table 2 and Table 3 summarize the antigenic targets and design strategies used in influenza virus-based vaccines located in the preclinical and clinical phases in the last 3 years, respectively. A total of nine generic influenza virus-based vaccines are in the preclinical stage and a total of two are in clinical trials. Most of these vaccines chose HA as the primary antigenic design target, and most of the design strategies were multi-target combination strategies, while combining strategies such as immune-focused strategies and AI to further enhance their broad spectrum, which highlights the importance of the HA target as well as the importance of combining different design strategies.

### 4.2. Universal Influenza Vaccine Based on Viral Vector

Viral vector-based vaccines use genetic engineering techniques to insert influenza antigenic targets into viral vectors, and when the recombinant virus infects host cells, the host cells express influenza antigens to induce an immune response. Adenovirus vectors, vesicular stomatitis virus (VSV) vectors, and poxviruses vectors are commonly used viral vectors. The adenoviral vector vaccine developed by the team at the Icahn School of Medicine at Mount Sinai and the University of Maryland, based on the HA of influenza H1, induced antibodies against the HA stem after a single dose in mice and provided 100% protection against homologous and heterologous pandemic H5 viruses [91], and could be used as a stockpile vaccine against pandemic influenza (Table 4). Interestingly, an influenza vaccine designed by a team from Tianjin Medical University and Fudan University using a chimpanzee adenovirus vector (AdC68) in combination with a multi-targeting strategy (multiple different influenza HA heads) and an immune-focusing strategy (one influenza HA stalk) induced high levels of antibody production against homologous pandemic H1N1, drifting seasonal H1N1, and H7N9 viruses and provided complete protection against these viruses [92] (Table 4). This ‘one stem to many heads’ approach protects against many different influenza subtypes and is an excellent addition to the universal influenza vaccine design. In addition, the St Jude Children’s Research Hospital team’s HA-based COBRA-designed adenovirus-vectored influenza vaccine not only induced broad-spectrum neutralizing antibodies after a single dose but also continued to provide complete protection against influenza viruses for at least 5 months after vaccination [93] (Table 4). Table 4 summarizes the antigenic targets and design strategies used for viral vector-based universal influenza vaccines in the preclinical phase in the last 3 years. A total of 16 viral vector-based universal influenza vaccines are in preclinical studies, and these vaccines have a more diverse selection of targets, including HA, NA, NP, M1, and PB1, combined with immunofocusing or T-cell strategies based on multi-targeted combination strategy design. A total of nine studies have adopted the T-cell strategy, which demonstrates the importance of the T-cell strategy in the design of universal vaccines. However, no new viral vector-based universal influenza vaccine has entered the clinical stage in the last 3 years.

### 4.3. Universal Influenza Vaccine Based on VLP

VLP-based vaccines are created by recombinantly expressing influenza virus antigenic proteins in appropriate expression systems (e.g., insect cell–baculovirus system, yeast cell system, etc.), which are capable of self-assembling to form particles similar to natural influenza viruses. VLP mimics the structure and spatial conformation of natural influenza viruses and can effectively stimulate a favorable immune response. The Sun Yat-sen University team designed and prepared influenza vaccines against influenza HA and NA in the form of VLP by mosaic algorithm, and the two VLP vaccines induced broader-spectrum neutralizing antibodies than the quadrivalent influenza vaccine in mice by mixing and injecting [107] (Table 5). Interestingly, the Georgia Institute of Technology and Icahn School of Medicine at Mount Sinai team developed a VLP vaccine with inverted HA that induced a broader immune response than the conventional vaccine [108] (Table 5). This suggests that it is possible to make the immunity focus on the HA stem by controlling the antigenic orientation. Table 5 summarizes the antigenic targets and design strategies used in VLP-based universal influenza vaccines located in the preclinical phase in the last 3 years. A total of 19 VLP-based universal influenza vaccines are in preclinical studies, and the antigenic targets of these vaccines are often not limited to HA, but also incorporate concurrent immune-focused or T-cell strategies based on multi-target co-design strategies, which again highlights the importance of combining multiple strategies. However, no new VLP-based universal influenza vaccine has entered the clinical phase in the last 3 years.

### 4.4. Universal Influenza Vaccine Based on Nanoparticles

Nanoparticle-based influenza vaccines are vaccines prepared by displaying influenza antigens onto nanoparticles by gene fusion technology or in vitro adhesion technology. The influenza vaccine prepared by the Chinese Academy of Sciences team by tandem linking HA stem epitopes, NP conserved epitopes, and M2e onto ferritin nanoparticles via a multi-targeting strategy induces a robust cross-reactive immune response and remains protective up to 6 months after vaccination [40] (Table 6). Interestingly, a vaccine prepared by the Georgia State University team by inserting influenza HA upside down into nanoparticles formed from extracellular vesicles protected mice from heterologous influenza viruses [126] (Table 6). This again suggests that adjusting the orientation of the antigen can focus the immune response to the stem of the HA. In addition, vaccines prepared by attaching antigenic targets of influenza to other nanoparticles such as PLGA [127], PLA [128], and hydrogels [129] similarly induced broad-spectrum and long-lasting immune responses (Table 6). This suggests that nanotechnology platforms can be one of the very promising platforms for universal influenza vaccine development. Table 3 and Table 6 summarize the antigenic targets and design strategies used for universal influenza vaccines based on nanoparticle platforms located in the preclinical and clinical phases in the last 3 years, respectively. A total of 34 generic nanoparticle-based influenza vaccines are in preclinical studies and a total of 4 are in clinical trials. The antigenic design targets of these vaccines are also not limited to HA, and most of them adopt multi-target combination strategies. Thirteen of these vaccines used an immunofocusing strategy, which highlights the advantages of immunofocusing strategies in the design of universal vaccines.

### 4.5. Universal Influenza Vaccine Based on Recombinant Proteins

Recombinant protein-based influenza vaccines do not require the expression of the full viral protein, but usually require only the recombinant expression of immunogenic proteins or parts of proteins in host cells. Such protein-based vaccines do not contain viral nucleic acids and usually have a favorable safety profile. An influenza protein vaccine prepared by the University of Georgia team against influenza HA or NA via the COBRA strategy induced neutralizing antibodies against seasonal influenza viruses isolated over the past 10 years [159,160,161] (Table 7). Interestingly, the Duke University team expressed multiple HA proteins through a multi-targeting strategy, and then injected mixtures of HA proteins into mice and ferrets to induce neutralizing antibodies against HA stems that were able to protect against homologous and heterologous influenza viruses [37] (Table 7). This suggests that mixtures of multiple HA proteins can partially overcome the immunodominance of the HA head. In addition, the China CDC team expressed recombinant protein vaccines with conserved peptides of HA, NA, and M2e through a multi-targeting strategy, which induced broad cross-protection against multiple influenza strains [162] (Table 7). This suggests that a protein vaccine of this peptide segment could also be one of the strong candidates for a universal influenza vaccine. Table 3 and Table 7 summarize the antigenic targets and design strategies used for recombinant protein-based universal influenza vaccines located in the preclinical and clinical stages in the last 3 years, respectively. A total of 16 recombinant protein-based universal influenza vaccines are in preclinical studies and a total of 3 are in clinical trials. These vaccines are more diverse in antigen selection, with more T-cell strategies based on multi-target combination strategies, which can compensate for the lack of cellular immunity in protein vaccines. The vaccines in the clinical stage all use HA as the antigen target, which also highlights the importance of the antigen target HA.

### 4.6. Universal Influenza Vaccine Based on Nucleic Acids

Nucleic acid-based influenza vaccines mainly include DNA vaccines and mRNA vaccines, which are designed to activate the immune response by injecting DNA sequences or mRNA sequences of specific influenza antigens into the body, respectively, and synthesizing viral proteins using the host’s transcription–translation system. An mRNA vaccine designed by the Chinese Academy of Sciences team based on influenza HA stems induced strong cellular and humoral immunity in mice and provided complete protection against homologous and heterologous influenza virus attacks [173] (Table 8). Interestingly, the NIAID and University of Pennsylvania teams used a multi-targeting strategy to prepare mRNA vaccines against 20 influenza HA subtypes, which induced broad-spectrum protection against both matched and unmatched influenza strains in mice and ferrets when injected in a mixture of these 20 mRNA vaccines [174] (Table 8). This may be the universal influenza mRNA vaccine capable of providing protection against the most diverse influenza subtypes at present; it also demonstrates that vaccines from mRNA technology platforms can induce antibodies against multiple antigens simultaneously. In addition, the University of Oslo team prepared a hybrid vaccine by designing the HA of 16 subtypes of influenza as DNA in the form of a heterodimer, and this hybrid form of the DNA vaccine induced T-cell responses and cross-immune responses against the conserved epitopes of the HA, as well as the production of neutralizing antibodies against the 2 subtypes of influenza that were not included [175] (Table 8). Table 3 and Table 8 summarize the antigenic targets and design strategies used in nucleic acid-based universal influenza vaccines located in the preclinical and clinical phases in the last 3 years, respectively. A total of 16 nucleic acid-based universal influenza vaccines are in preclinical studies and a total of 15 are in clinical trials. These vaccines often do not have a single antigenic target but rather use a multi-target combination strategy in conjunction with other strategies such as the T-cell and COBRA strategies. This also highlights the importance of using multiple targets and multiple design strategies in universal vaccine design.

## 5. Conclusions and Perspective

The development of a universal influenza vaccine is of significant public health importance. The last three years have seen great progress in the development of universal influenza vaccines. Many new techniques such as antigenic mutant libraries [37] and inverted HA [108,126] have been used to focus the immune response to conserved stems, as well as multi-targeted combination strategies to couple multiple antigens into the same nanocarrier to provoke a broader-spectrum immune response than mixing them individually [38]. The use of equine-origin influenza as a vaccine strain may reduce immune imprinting while increasing the vaccine’s broad spectrum [54], which suggests that we can go beyond human sources in vaccine immunogen selection. The six vaccine development platforms mentioned above can all contribute to the development of a universal influenza vaccine through the use of these emerging vaccine design technologies. Although there is no approved universal influenza vaccine yet, the continuous development and application of new technologies will accelerate the development and launch of universal influenza vaccines.

## Figures and Tables

**Table 1:** Five common targets for universal influenza vaccines.

Targets	Structure and Functions	Immune Responses
HA	Type I transmembrane glycoproteins	HA-conjugated antibodies
	Homotrimeric forms	Anti-HA head antibodies: strain-specific
	Monomers are divided into head and stem	Anti-HA stem antibodies: cross-protection, ADCC
	Binds to sialic acid receptors	
NA	Type II transmembrane glycoproteins	NA-conjugated antibodies
	Homotetrameric forms	Anti-NA antibody: cross-protection
	Promoting the release of viruses	
M2	Type III transmembrane proteins	M2e-conjugated antibodies
	Homotetrameric forms	Anti-M2e antibodies: cross-protection, ADCC
	Guiding viruses into and out of the cell	
NP, M1	Internal proteins of the virus	Cross-protection
	M1: the formation of viral particles	T-cell response
	NP: viral replication-related	

Notes: HA, haemagglutinin; NA, neuraminidase; NP, nuclear protein; M1, matrix protein 1; M2, matrix protein 2; M2e, M2 extracellular domains; ADCC, antibody-dependent cell-mediated cytotoxicity.

**Table 2:** Universal influenza vaccines based on influenza virus technology platforms in preclinical stage.

Time	Name	Developer	Antigen Targets	Design Strategies	Reference
2025	hlHA IIV	Duke University (US)	Whole virus proteins based on HA stem	Immunofocusing strategy and multi-target combination strategy	[53]
2025	H1, H3 COBRA IIV	University of Georgia (US) and Cleveland Clinic (US)	Whole virus proteins based on HA	COBRA strategy and multi-target combination strategy	[59]
2025	CLEARFLU	University of Oxford (UK) and University of Melbourne (Australia)	Whole virus proteins based on HA	Multi-target combination strategy	[60]
2024	H3N8 live attenuated virus vaccine	Iowa State University (US)	Whole virus proteins	Multi-target combination strategy	[54]
2024	HA-based whole inactivated virus	Icahn School of Medicine at Mount Sinai (US)	Whole virus proteins based on HA	COBRA strategy and multi-target combination strategy	[61]
2024	ΔNS1 virus	Icahn School of Medicine at Mount Sinai (US)	Whole virus proteins based on HA	Immunofocusing strategy and multi-target combination strategy	[62]
2023	Reassortant LAIV with modified NS-1 and NP	Institute of Experimental Medicine (Russia)	Whole virus proteins based on NP	Multi-target combination strategy and T-cell strategy	[63]
2022	PROTAR	Chinese Academy of Sciences (China)	M1 ubiquitinated whole viral proteins	Multi-target combination strategy	[55]
2022	NAe-HA and M2e-HA	Georgia State University (US)	Whole virus proteins based on HA, NA, and M2e	Multi-target combination strategy	[64]

Notes: Influenza virus technology includes activated and inactivated influenza virus vectors that are used as backbones for expression of relevant influenza antigens.

**Table 3:** Universal influenza vaccines in clinical stage under different technological routes.

Technological Routes	Start Time	Name	Developer	Clinical Phase, Registry ID	Antigen Targets	Design Strategies	References
Influenza-virus	2022	CodaVax	Codagenix (US)	Phase I, NCT05223179	Whole virus proteins based on HA and NA	Multi-target combination strategy and artificial intelligence strategy	[65,66,67]
	2022	BPL-1357	NIAID (US)	Phase I, NCT05027932	Whole virus proteins (H7N3, H5N1, H3N8, and H1N9)	Multi-target combination strategy	[68]
Nanoparticles	2025	FluMos self-assembling nanoparticle	NIAID (US)	Phase I, NCT06863142	HA	T-cell strategy	[69,70,71]
	2024	OVX836	Osivax (France)	Phase II, NCT06582277	Multiple types of NP	Multi-target combination strategy	[72,73]
	2024	CIC Vaccine	Novavax (US)	Phase III, NCT06482359	Multiple types of HA	Multi-target combination strategy	Withdrawn
	2024	Nano-Flu (tNIV)	Novavax (US) and Emergent BioSolutions (US)	Phase III, NCT06485752	Multiple types of HA	Multi-target combination strategy	[74,75]
Recombinant protein	2024	RIV3 + NVXC19	Sanofi (France)	Phase II, NCT06695130	3 types of HA	Multi-target combination strategy	Results not yet reported
	2024	TIV-HD + NVXC19 Combination vaccine	Sanofi (France)	Phase II, NCT06695117	3 types of HA	Multi-target combination strategy	Results not yet reported
	2023	G1 mHA	Janssen Vaccines and Prevention, J&J (Netherlands)	Phase II, NCT05901636	HA stem	Immunofocusing strategy	[35,76,77,78,79]
Nucleic acid	2025	mRNA constructs	Sanofi (France)	Phase II, NCT06744205	1 or 4 or 6 types NA	Multi-target combination strategy	[80]
	2024	sa-RNA (ARCT-2138)	Arcturus Therapeutics (US) and CSL Seqirus (Australia)	Phase I, NCT06602531	4 types of HA	Multi-target combination strategy	Results not yet reported
	2024	mRNA Flu/COVID-19 combination vaccine	GSK (UK) and CureVac (Germany)	Phase II, NCT06680375	4 types of HA	Multi-target combination strategy	Results not yet reported
	2024	mRNA-1010	Moderna (US)	Phase III, NCT06602024	4 types of HA	Multi-target combination strategy	[81]
	2024	mRNA-1083	Moderna (US)	Phase III, NCT06694389	4 types of HA	Multi-target combination strategy	[82]
	2023	Multivalent modified mRNA	GSK (UK) and CureVac (Germany)	Phase II, NCT06431607	4 types of HA	Multi-target combination strategy	[83]
	2023	mRNA-1011 and mRNA-1012	Moderna (US)	Phase II, NCT05827068	5 or 6 types of HA	Multi-target combination strategy	[84]
	2023	H1ssF_3928 mRNA-LNP	CIVICs, NIAID (US)	Phase I, NCT05755620	HA stem	Immunofocusing strategy	Results not yet reported
	2023	sa-mRNA (SQ012)	CSL Seqirus (Australia)	Phase I, NCT06028347	HA and NA	Multi-target combination strategy	[85,86,87]
	2023	DCVC H1 HA mRNA-LNP	NIAID (US)	Phase I, NCT05945485	HA	No	Results not yet reported
	2023	modRNA-based combination	Pfizer (US) and BioNTech (Germany)	Phase III, NCT06178991	4 types of HA	Multi-target combination strategy	[88]
	2022	mRNA-1230	Moderna (US)	Phase I, NCT05585632	4 types of HA	Multi-target combination strategy	Results not yet reported
	2022	mRNA-1020 and mRNA-1030	Moderna (US)	Phase II, NCT05333289	HA and NA	Multi-target combination strategy	[89]
	2022	saRNA	Pfizer (US)	Phase II, NCT05227001	HA and NA	Multi-target combination strategy	Results not yet reported
	2022	Modified mRNA vaccine	Pfizer (US)	Phase III, NCT05540522	4 types of HA	Multi-target combination strategy	[90]

Notes: ‘No’ means that the study did not use any of the broad-spectrum vaccine design strategies discussed in this article, and only wild-type full-length HA was used as the immunogen.

**Table 4:** Universal influenza vaccines based on viral vector in preclinical stage.

Time	Name	Developer	Antigen Targets	Design Strategies	Reference
2025	HAdV5-HNH	China CDC (China)	HA stem and NA	Multi-target combination strategy and immunofocusing strategy	[94]
2025	AdC-Flu-Tet	Fudan University (China)	HA, NP, and M2e	Multi-target combination strategy and T-cell strategy	[95]
2025	Ad vector-based vaccine with autophagy-inducing peptide	Purdue University (US)	HA stem	Immunofocusing strategy	[96]
2025	Epigraph HA	University of Nebraska−Lincoln (US)	Multiple types of HA	Multi-target combination strategy	[97]
2024	rAd/NP + rAd/HA-M2e	Ewha Womans University (Korea)	HA, NA, and M2e	Multi-target combination strategy and T-cell strategy	[98]
2024	rAd-NP-M2e-GFP	Jilin University (China)	NP and M2e	Multi-target combination strategy and T-cell strategy	[99]
2024	CyCMV/Flu	Oregon Health & Science University (US)	NP, M1, and PB1	Multi-target combination strategy and T-cell strategy	[100]
2024	ChAdOx2-NPM1-NA2 and MVA-NPM1NA2	Pirbright Institute (UK)	NP and M1	Multi-target combination strategy and T-cell strategy	[101]
2024	rAAV-COBRA	St Jude Children’s Research Hospital (US)	HA	COBRA strategy	[93]
2024	AdC68-cHAs	Tianjin Medical University (China) and Fudan University (China)	Multiple types of HA	Multi-target combination strategy and immunofocusing strategy	[92]
2024	rVSV-EΔM-tM2e	University of Manitoba (Canada)	Multiple types of M2e	Multi-target combination strategy	[102]
2023	A/NP + M2-rAd	Food and Drug Administration (US)	NP and M2	Multi-target combination strategy and T-cell strategy	[103]
2023	MVA-NP	German Center of Infection Research (DZIF) (Germany)	NP	T-cell strategy	[104]
2023	Wyeth/IL-15/5flu	University of Hong Kong (Hong Kong SAR, China)	HA, NA, NP, M1, and M2	Multi-target combination strategy and T-cell strategy	[105]
2022	rMVA-k1-k2	Federal Medical-Biological Agency (Russia)	HA, NP, and M1	Multi-target combination strategy and T-cell strategy	[106]
2022	Ad-5-H1	Icahn School of Medicine at Mount Sinai (US) and University of Maryland (US)	HA	Immunofocusing strategy	[91]

**Table 5:** Universal influenza vaccines based on VLP in preclinical stage.

Time	Name	Developer	Antigen Targets	Design Strategies	Reference
2025	H3N1M2e5x VLP	Kyung Hee University (S Korea)	HA, NA, and M2e	Multi-target combination strategy	[109]
2025	NA-M2e VLP	Georgia State University (US)	NA and M2e	Multi-target combination strategy	[110]
2024	cVLPs	University of Copenhagen (Denmark) and Scripps Research Institute (US)	HA stem and NA	Multi-target combination strategy and immunofocusing strategy	[111,112]
2024	COBRA-VLP	University of Georgia (US) and Cleveland Clinic (US)	NA	COBRA strategy	[48]
2024	N2-VLPs	University of Natural Resources and Life Sciences Vienna (BOKU) (Austria)	HA and NA	Multi-target combination strategy	[113]
2024	PR8HA-VLP	University of Wisconsin (US) and Georgia Institute of Technology (US)	HA	Multi-target combination strategy	[114]
2024	HBc VLPs	Chinese Academy of Sciences (China)	NP and M2e	Multi-target combination strategy and T-cell strategy	[115]
2024	Cap-Cat VLPs	Henan Academy of Agricultural Sciences (China) and Zhengzhou University (China)	3 types of M2e	Multi-target combination strategy	[116]
2024	Quadrivalent VLPs	National Health Research Institutes (Taiwan)	HA, NA, and M1	Multi-target combination strategy and T-cell strategy	[117]
2023	Inverted HA VLP	Georgia Institute of Technology (US) and Icahn School of Medicine at Mount Sinai (US)	HA	Immunofocusing strategy	[108]
2023	HA-VLP-Cyt	Georgia State University (US)	HA and M1	Multi-target combination strategy and T-cell strategy	[118]
2023	cVLPs	Jiaxing University (China)	HA stem and M2e	Multi-target combination strategy and immunofocusing strategy	[119]
2023	M2e VLP MP	Mercer University (US)	Multiple types of M2e	Multi-target combination strategy	[120]
2023	Mosaic VLPs	Sun Yat-sen University (China)	HA and NA	Multi-target combination strategy and T-cell strategy	[107]
2022	NA2 VLP	Auburn University (US) and Emory-UGA CEIRS (US)	NA and M1	Multi-target combination strategy and T-cell strategy	[121]
2022	Hybrid fusion protein combination vaccine	Emory University (US) and Georgia State University (US)	HA and M1	Multi-target combination strategy and T-cell strategy	[122]
2022	NA-VLPs	King Mongkut’s University of Technology Thonburi (Thailand)	NA	Immunofocusing strategy	[123]
2022	Chimeric cytokine HA-VLP vaccine	Nerome Institute of Biological Resources (Japan)	NA and M2	Multi-target combination strategy	[124]
2022	SpyTagged noro-VLP	Tampere University (Finland)	HA and M2e	Multi-target combination strategy and immunofocusing strategy	[125]

**Table 6:** Universal influenza vaccines based on nanoparticle platform in preclinical stage.

Time	Name	Developer	Antigen Targets	Design Strategies	Reference
2025	LBL HA-4M2e NPs	Georgia Institute of Technology (US)	HA and M2e	Multi-target combination strategy	[130]
2025	Inverted HA-extracellular vesicles (EVs)	Georgia State University (US)	HA	Immunofocusing strategy	[126]
2025	HA2-16 ferritin nanoparticles	Jilin University (China)	HA stem	Immunofocusing strategy	[131]
2025	CHM-f nanoparticle	Northwest A&F University (China) and Chengdu NanoVAX Biotechnology (China)	HA and M2e	Multi-target combination strategy	[132]
2025	Ferritin-HA	Shanghai Institute of Biological Products (China)	HA	Multi-target combination strategy	[133]
2025	Adjuvanted PNP-hydrogel system	Stanford University (US)	Multiple types of HA	Multi-target combination strategy	[129]
2024	IAV-nanovax	University of Iowa (US) and Iowa State University (US)	HA and NP	Multi-target combination strategy and T-cell strategy	[134]
2024	BP-NP366/PA224	University of Melbourne (Australia) and Griffith University (Australia)	NP and PA	Multi-target combination strategy and T-cell strategy	[135]
2024	TMV-HA	University of Nebraska−Lincoln (US)	HA	T-cell strategy	[136]
2024	3M2e-R4R5	University of Quebec at Montreal (Canada)	Multiple types of M2e	Multi-target combination strategy	[137]
2024	NA-Mi3 nanoparticles	Utrecht University (Netherlands) and Ghent University (Belgium)	Multiple types of NA	Multi-target combination strategy	[138]
2024	HA-SAV	Yonsei University (South Korea)	HA	Multi-target combination strategy	[139]
2024	NM2e@DDAB/PLA nanovaccine	Chinese Academy of Sciences (China)	NP and M2e	Multi-target combination strategy and T-cell strategy	[128]
2024	Double-layered protein nanoparticles	Georgia State University (US)	HA stem, NP, and M2e	Multi-target combination strategy, immunofocusing strategy, and T-cell strategy	[140]
2024	HA/GP nanoparticles	Georgia State University (US)	HA	Multi-target combination strategy	[141]
2024	Self-assembled multiepitope nanoparticles (MHF)	Jilin University (China)	HA stem, NP, and M2e	Multi-target combination strategy, immunofocusing strategy, and T-cell strategy	[142]
2024	Self-assembling protein nanoparticles (SApNPs)	Scripps Research Institute (US)	Multiple types of M2e	Multi-target combination strategy	[143]
2024	Hexaplex liposomes	State University of New York at Buffalo (US)	HA and NA	Multi-target combination strategy	[144]
2024	M2e Nanoclusters	Texas Tech University (US) and Georgia State University (US)	Multiple types of M2e	Multi-target combination strategy	[145]
2023	3M2e-rHF nanoparticle	Chinese Academy of Sciences (China)	HA, NA, and M2e	Multi-target combination strategy	[40]
2023	Self-assembled protein nanocages	Georgia Institute of Technology (US)	HA stem and M2e	Multi-target combination strategy and immunofocusing strategy	[146]
2023	ISCOMs/MPLA-adjuvanted SDAD protein nanoparticles	Georgia State University (US)	NP, M2e, and NA	Multi-target combination strategy and T-cell strategy	[147]
2023	3M2e-T4 nanoparticle	Huazhong Agricultural University (China)	Multiple types of M2e	Multi-target combination strategy	[148]
2023	Combinatorial polymeric nanoshell	National Taiwan University (China)	M2e	Immunofocusing strategy	[149]
2023	Helix-A stem nanoparticle	NIAID (US)	HA stem	Immunofocusing strategy	[150]
2023	PLGA nanoparticles	Sunway University (Malaysia)	M2 and NP	Multi-target combination strategy and T-cell strategy	[127]
2023	Multivalent DNA nanovaccine	Taizhou University (China)	HA and NA	Multi-target combination strategy	[151]
2022	M2e/CpG-ODN/TMC	Agricultural Research, Education and Extension Organization (Iran)	M2e	Immunofocusing strategy	[152]
2022	Mini-HA-LS Nano-vaccine	Huazhong Agricultural University (China)	HA stem	Immunofocusing strategy	[153]
2022	Adjuvanted nanoparticle fusion constructs	Indian Institute of Science (India)	HA stem	Immunofocusing strategy	[154]
2022	3MCD-f nanovaccine	Jilin University (China)	HA stem and M2e	Multi-target combination strategy and immunofocusing strategy	[155]
2022	CDh-f nanoparticle	Jilin University (China)	HA stem	Immunofocusing strategy	[156]
2022	Self-assembling peptides displaying M2e and HA2	Russian Academy of Sciences (Russia)	HA stem and M2e	Multi-target combination strategy and immunofocusing strategy	[157]
2022	CTA1-3M2e-DD (FPM2e)	University of Gothenburg (Sweden) and Ghent University (Belgium)	Multiple types of M2e	Multi-target combination strategy	[158]

**Table 7:** Universal influenza vaccines based on recombinant protein in preclinical stage.

Time	Name	Developer	Antigen Targets	Design Strategies	Reference
2025	P125-H	China CDC (China)	HA, NA, and M2	Multi-target combination strategy	[162]
2025	Mosaic nucleoprotein (MNP)	University of Wisconsin (US)	NP	T-cell strategy	[43]
2025	Recombinant HA proteins	University of Wisconsin (US)	2 types of HA	Multi-target combination strategy	[163]
2024	Sbmut HA	Duke University (US)	Multiple types of HA	Multi-target combination strategy and immunofocusing strategy	[37]
2024	M2e-H3 stalk	Georgia State University (US)	HA and M2e	Multi-target combination strategy	[164]
2024	M2e-based recombinant fusion proteins	Russian Ministry of Health (Russia)	HA, NP, and M2e	Multi-target combination strategy and T-cell strategy	[165]
2024	rTET-NA	Sanofi (France)	HA and NA	Multi-target combination strategy	[166]
2024	HAm	Sun Yat-sen University (China)	HA	T-cell strategy	[167]
2024	COBRA HA proteins	University of Georgia (US)	HA	COBRA strategy	[159]
2024	COBRA H1 HA	University of North Carolina (US) and University of Georgia (US)	HA	COBRA strategy	[161]
2023	Scrambled HA (scrHA)	University of Wisconsin (US)	HA	Immunofocusing strategy	[168]
2023	LHNVD-105/110	Longhorn Vaccines and Diagnostics (US)	HA, NA, and M2e	Multi-target combination strategy	[169]
2023	3M2e-HA2-NP chimeric subunit	Pasteur Institute of Iran (Iran)	HA, NP, and M2e	Multi-target combination strategy and T-cell strategy	[170]
2023	N1-I COBRA NA antigen	University of Georgia (US)	NA and HA	COBRA strategy and multi-target combination strategy	[160]
2022	B60-Stem-8071	Xiamen University (China)	HA stem	Immunofocusing strategy	[171]
2022	rM2e-ΔPly	Chongqing Medical University (China)	3 types of M2e	Multi-target combination strategy	[172]

**Table 8:** Universal influenza vaccines based on nucleic acids in preclinical stage.

Time	Name	Developer	Antigen Targets	Design Strategies	Reference
2025	5xM2e mRNA lipid nanoparticle	Georgia State University (US)	5 types of M2e	Multi-target combination strategy	[176]
2024	Mosaic NA1 (mNA1)	Sun Yat-sen University (China)	NA	T-cell strategy	[177]
2024	NA-targeting circRNA vaccine	Sun Yat-sen University (China)	3 types of NA	Multi-target combination strategy	[178]
2024	COBRA HA-encoding mRNA	University of Georgia (US)	2 types of HA	Multi-target combination strategy and COBRA strategy	[179]
2024	HA-encoding mRNA	University of Georgia (US)	4 types of HA	Multi-target combination strategy	[180]
2024	mRNA LNP vaccine encoding a Y2 COBRA HA immunogen	University of North Carolina (US) and University of Georgia (US)	HA	COBRA strategy	[181]
2024	Multiple HA-DNA	University of Oslo (Norway)	16 types of HA	Multi-target combination strategy	[175]
2024	HA, NP, and 3M2e mRNA	Chinese PLA General Hospital (China)	HA, NP, and M2e	Multi-target combination strategy and T-cell strategy	[182]
2024	NA-F2A-HA mRNA-LNP	Duke University (US)	HA and NA	Multi-target combination strategy	[39]
2024	dbDNA-encoded NA	Imperial College London (UK)	HA and NA	Multi-target combination strategy	[183]
2024	Multi-epitope mRNA-based vaccines	Key Laboratory of Jilin Province for Zoonosis Prevention and Control (China)	HA and M2e	Multi-target combination strategy and T-cell strategy	[184]
2023	MLN-mRNA	Shanghai Institute of Biological Products (China)	HA stem, M2e, and NP	Immunofocusing strategy and multi-target combination strategy	[41]
2023	mHAs	Chinese Academy of Sciences (China)	HA stem	Immunofocusing strategy	[173]
2023	Quadrivalent HA mRNA	Greenlight Biosciences (US) Name	4 types of HA	Multi-target combination strategy	[185]
2022	mRNA/LNP vaccine	Merck & Co. (US)	HA stem and NP	Immunofocusing strategy and multi-target combination strategy	[186]
2022	mRNA-Flu	National Institute for Public Health and the Environment (Netherlands)	NP, M1, and PB1	Multi-target combination strategy and T-cell strategy	[45]
2022	20 mRNA-LNP	CIVICs, NIAID (US) and University of Pennsylvania (US)	20 types of HA	Multi-target combination strategy	[174]
2022	cGAMP-adjuvanted multivalent mRNA vaccines	Georgia State University (US)	2 types of HA, M1, and NP	Multi-target combination strategy and T-cell strategy	[187]
